# Evaluating Hyponatremia as a Predictor of Mortality in Heart Failure Patients in a Tertiary Healthcare Setting

**DOI:** 10.7759/cureus.96048

**Published:** 2025-11-03

**Authors:** Obaidullah Durrani, Shumail Saeed, Shumaila Kanwel, M Khaliq, Ayesha Farrukh

**Affiliations:** 1 Orthopaedics, Sargodha Medical College, Sargodha, PAK; 2 Medical Imaging, Riphah International University, Islamabad, PAK; 3 Pharmacology, Abu Umara Medical and Dental College, Lahore, PAK; 4 Pathology, Federal Postgraduate Medical Institute, Lahore, PAK; 5 Pathology, University of Health Sciences, Lahore, PAK; 6 Health Sciences, Panjab University, Lahore, PAK; 7 Medical School, Rawalpindi Medical University, Rawalpindi, PAK

**Keywords:** complications, heart failure, hyponatremia, mortality, prognosis, risk factors, serum sodium

## Abstract

Background

Heart failure (HF) is a condition that has a high morbimortality, with high levels of hospitalization and death in most countries. Hyponatremia is a frequent electrolyte imbalance in HF and can indicate neurohormonal release and disease severity. This study determined the effects of admission hyponatremia on in-hospital and 30-day mortality in hospitalized HF patients.

Methodology

In this prospective cohort study, 220 patients with HF admitted to a tertiary cardiology unit from September to December 2023 were enrolled. Patients were classified into hyponatremic (<135 mmol/L), mild (130-134 mmol/L), moderate-to-severe (<130 mmol/L), and normonatremic (≥135-145mmol/L). The severity of HF was determined as left ventricular ejection fraction (LVEF <40%, 40-49%, and ≥50%). Demographics, comorbidities, renal function, and medications were documented. Independent t-tests and chi-square tests were used for statistical analysis, with p-values <0.05 considered significant. Cox regression models were used to assess mortality outcomes after adjusting for renal function and diuretic class.

Results

Hyponatremia was found in 92 (41.8%) patients, more common in cases with low LVEF. Higher in-hospital mortality (17 (18.5%) vs 9 (7.0%), p = 0.011), 30-day mortality (23 (25.0%) vs. 15 (11.7%), p = 0.004) were related to hyponatremia compared with normonatremia (9 (7.0%), p = 0.004). The level of mortality increased as sodium levels worsened. An adjusted Cox analysis showed that there was a relationship between hyponatremia and mortality (hazard ratio = 2.1; 95% confidence interval = 1.2-3.7; p = 0.008).

Conclusions

Admission hyponatremia is associated with higher in-hospital and 30-day mortality in HF patients, particularly those with low ejection fraction. Regular sodium monitoring and prompt correction can enhance early risk stratification and short-term outcomes.

## Introduction

Heart failure (HF) has a high morbidity and mortality, with high levels of hospitalization and death in most countries, impacting more than 60 million people and posing a significant healthcare burden and cost to the economy [1]. Although new pharmacological and device-based therapies have been developed, the prognosis of hospitalized patients with HF remains poor, and the in-hospital mortality and the readmission rates are unacceptable [2]. This demonstrates the requirement of dependable, user-friendly, and affordable prognostic variables to enhance the initial stratification of risks and manage them. Numerous studies have highlighted that basic biochemical markers, especially serum sodium levels, might act as significant prognostic indicators in the acute circumstances of HF.

One of the most common electrolyte imbalances in HF is hyponatremia, which is defined as a serum sodium level less than 135 mmol/L, and is estimated to have a prevalence rate between 20% and 30% [3]. This demonstrates neurohormonal stimulation, especially the non-osmotic release of arginine vasopressin, upregulation of the renin-angiotensin-aldosterone system (RAAS), and sympathetic stimulation that culminate in water retention and hemodynamic instability [4]. Previous studies have suggested that hyponatremia can be a marker of disease severity and an important risk factor associated with adverse outcomes, such as longer hospitalization, high readmission rates, and increased short- and long-term mortality [5]. Recent meta-analyses have substantiated this correlation and emphasized that low sodium levels indicate neurohormonal dysregulation and fluid overload, rather than serving as a direct cause of mortality. Additionally, the extent of HF may affect the prognostic value of hyponatremia, especially in patients with a lower ejection fraction, which indicates more advanced ventricular dysfunction.

Nevertheless, hyponatremia has a questionable prognostic value. According to previous research, it is mostly a sign of a progressive illness and does not play a significant role in risk, and its consequences depend on comorbidities, therapy, and demographic differences [6]. Additionally, the majority of available evidence is based on the Western population, whereas the data from South Asia and developing countries are limited [7]. Given the increasing prevalence of HF in these areas, the detection of prognostic factors such as hyponatremia is of great clinical importance [8].

The objective of the current research was to assess the incidence of hyponatremia among hospitalized HF patients and identify it as an independent factor of in-hospital and short-term mortality in the South Asian population.

## Materials and methods

This prospective cohort study was performed at the Cardiology Department of a tertiary healthcare facility associated with the University of the Punjab, Lahore, from September to December 2023 (approval number: 147/09/2023). The aim was to ascertain whether admission hyponatremia is associated with in-hospital and 30-day mortality in hospitalized HF patients. Adult patients (≥18 years) diagnosed with HF were consecutively reenrolled using a non-probability sampling approach after obtaining written informed consent. The sample size (n = 220) was determined utilizing OpenEpi version 3.0.0 (Atlanta, GA, USA) at a 95% confidence level, with a 5% margin of error and an estimated prevalence of hyponatremia at 40% [9].

According to the European Society of Cardiology (ESC) 2021 criteria, HF severity was classified as mild (left ventricular ejection fraction (LVEF) ≥50%), moderate (40-49%), or severe (<40%). We used the ESC 2021 guidelines because they provide standardized diagnostic criteria for HF, including clinical symptoms, physical findings, and echocardiographic evidence of structural or functional cardiac abnormality. The criteria for inclusion were confirmed HF, age at least 18 years old, and having baseline sodium and renal function data available within 24 hours of admission. Patients with acute coronary syndrome, chronic kidney disease (CKD) stage IV-V, acute infection, hepatic cirrhosis, pregnancy, endocrine causes of hyponatremia (e.g., syndrome of inappropriate antidiuretic hormone secretion, adrenal insufficiency), or a major diuretic dose change within the preceding 48 hours were excluded.

An ion-selective electrode analyzer was used to measure serum sodium and osmolality within 24 hours of admission, with daily internal quality control procedures performed. Normonatremia was characterized by levels ranging from 135 to 145 mmol/L, while hyponatremia was identified at levels below 135 mmol/L, further classified into mild (130-134 mmol/L) and moderate-to-severe (<130 mmol/L) categories. Serum creatinine, estimated glomerular filtration rate (estimated glomerular filtration rate, CKD-EPI formula), and acute kidney injury stage per the Kidney Disease: Improving Global Outcomes criteria were used to assess the baseline renal function. Medication histories, such as loop and thiazide diuretics, RAAS inhibitors, beta-blockers, and angiotensin receptor-neprilysin Inhibitors, were recorded, and their classes and dosages were noted. The time to event (in-hospital and 30-day mortality) was identified with the help of Kaplan-Meier survival curves and log-rank tests and was censored in case of patients who were discharged alive or survived within 30 days. Multivariate Cox proportional hazards regression analyses were developed to determine the relationship between hyponatremia and mortality after accounting for age, sex, LVEF, body mass index (BMI), renal function, and diuretic class. Schoenfeld residuals were used to check the assumptions of proportional hazards.

SPSS version 26.0 (IBM Corp., Armonk, NY, USA) was used for statistical analyses. t-tests were used to compare continuous variables, and chi-square tests were used to compare categorical variables. Cox proportional hazards and logistic regression models were utilized to assess the correlation between hyponatremia and mortality outcomes. The findings are presented as hazard ratios (HRs) accompanied by a 95% confidence interval (CI). Mortality and sodium missing data were eliminated, and complete case analysis was used for other variables with <5% missing values. Clinical records and telephone confirmation confirmed that the 30-day follow-up was 98% complete.

## Results

A total of 220 patients admitted to the hospital with HF were selected to test the association between the severity of hyponatremia and mortality. Table 1 presents the demographic and clinical features of patients according to the severity of hyponatremia at the time of admission. Of the 220 patients admitted to the hospital with HF, 128 (58.2% normonatremic) and 92 (41.8% hyponatremic) patients were found to be mild, moderate, and severe, respectively. Another finding was that a progressive reduction in mean LVEF and a progressive increase in BMI were linked to increasing severity of hyponatremia. There were no statistically significant differences in the sodium groups regarding age, sex distribution, hypertension, and diabetes mellitus.

**Table 1:** Demographic and clinical characteristics of hospitalized HF patients (n = 220). *: Significant at p-values <0.05. n = number of participants; SD = standard deviation; BMI = body mass index; LVEF = left ventricular ejection fraction; eGFR = estimated glomerular filtration rate; RAAS = renin-angiotensin-aldosterone system; ANOVA = analysis of variance; HF = heart failure

Parameter	Total (n = 220)	Normonatremia (≥135 mmol/L; n = 128)	Mild (130–134 mmol/L; n = 50)	Moderate (125–129 mmol/L; n = 25)	Severe (<125 mmol/L; n = 17)	Statistical test	Test value	P-value
Age (years, mean ± SD)	61.2 ± 11.3	60.0 ± 11.6	61.5 ± 10.5	63.0 ± 11.0	64.0 ± 12.0	ANOVA	1.82	0.144
Male, n (%)	138 (62.7%)	83 (64.8%)	30 (60%)	15 (60%)	10 (59%)	Chi-square	0.95	0.813
Female, n (%)	82 (37.3%)	45 (35.2%)	20 (40%)	10 (40%)	7 (41%)	Chi-square	0.95	0.813
BMI (kg/m², mean ± SD)	27.9 ± 4.5	27.4 ± 4.4	28.2 ± 4.5	29.0 ± 4.8	29.5 ± 4.9	ANOVA	3.05	0.029*
LVEF (%, Mean ± SD)	35.8 ± 9.1	37.8 ± 9.0	35.0 ± 8.5	32.5 ± 9.0	31.0 ± 8.5	ANOVA	8.23	<0.001*
Hypertension, n (%)	148 (67.3%)	84 (65.6%)	35 (70%)	18 (72%)	11 (65%)	Chi-square	0.74	0.863
Diabetes mellitus, n (%)	106 (48.2%)	56 (43.8%)	25 (50%)	14 (56%)	11 (65%)	Chi-square	2.31	0.509
Serum creatinine (mg/dL, mean ± SD)	1.26 ± 0.32	1.12 ± 0.20	1.22 ± 0.25	1.38 ± 0.30	1.52 ± 0.35	ANOVA	14.5	<0.001*
eGFR (mL/min/1.73m², mean ± SD)	77 ± 18	85 ± 15	75 ± 16	66 ± 14	59 ± 12	ANOVA	22.1	<0.001*
AKI present, n (%)	42 (19%)	12 (9%)	10 (20%)	12 (48%)	8 (47%)	Chi-square	27.4	<0.001*
Loop/Thiazide diuretics, n (%)	110 (50%)	45 (35%)	25 (50%)	20 (80%)	20 (85%)	Chi-square	35.2	<0.001*
RAAS inhibitors, n (%)	140 (64%)	85 (66%)	30 (60%)	15 (60%)	10 (59%)	Chi-square	1.2	0.75

Worsening of cardiac functioning and increased BMI were linked to progressive hyponatremia in patients admitted with HF. Mean LVEF decreased gradually between normonatremic patients (37.8 ± 9.0) and mild (35.0 ± 8.5), moderate (32.5 ± 9.0), and severe hyponatremia (31.0 ± 8.5). Likewise, sodium strata had a rise in BMI (27.4 ± 4.4 to 29.5 ± 4.9 kg/m²; p = 0.029; SDM = 0.32). There were no considerable differences in terms of age, sex, hypertension, or diabetes. These results suggest that there is a progressive connection between serum sodium drop and unfavorable clinical phenotype, and sodium concentration may be an effective indicator of disease severity in HF. Table 2 shows the relationship between serum sodium status and patient outcomes.

**Table 2:** Sodium status and patient outcomes by hyponatremia severity. *: Significant at p-values <0.05. Cox regression models for mortality were adjusted for clinical parameters (LVEF, BMI, Creatinine, eGFR). In-hospital mortality counts only patients who died in the hospital, 30-day mortality counts patients who died within 30 days, including both in-hospital deaths and deaths after discharge. As per standard generalizability concerns, only the events were reported; the remaining patients in each category who did not experience mortality were considered survivors. LVEF = left ventricular ejection fraction; BMI = body mass index; eGFR = estimated glomerular filtration rate; ANOVA = analysis of variance

Variable	Total (n = 220)	Normonatremia (≥135 mmol/L; n = 128)	Mild (130–134 mmol/L; n = 50)	Moderate (125–129 mmol/L; n = 25)	Severe (<125 mmol/L; n = 17)	Statistical test	Test value	P-value
Serum sodium (mmol/L, mean ± SD)	136.1 ± 4.8	139.4 ± 2.1	132.2 ± 1.2	127.5 ± 1.4	123.0 ± 1.5	ANOVA	182.5	<0.001*
In-hospital mortality, n (%)	27 (12.3%)	9 (7.0%)	5 (10%)	6 (24%)	6 (35%)	Chi-square	18.2	<0.001*
30-day mortality, n (%)	40 (18.2%)	15 (11.7%)	8 (16%)	7 (28%)	8 (47%)	Chi-square	19.6	<0.001*
Mean hospital stay (days ± SD)	7.8 ± 3.6	6.8 ± 3.2	8.5 ± 3.5	9.5 ± 4.0	10.2 ± 4.2	ANOVA	9.1	<0.001*

There was a positive relationship between falling serum sodium and bad outcomes of a graded nature. Hospital death increased to 35% in severe hyponatremia and 7.0% in normonatremic patients, whereas 30-day mortality went up to 47% and 11.7%, respectively (p < 0.001). The mean hospital stay increased to 10.2 (SD = 4.2) days. These results demonstrate that there is an evident dose-response association between the degree of hyponatremia and short-term mortality among HF patients. Table 3 shows the Cox regression analysis demonstrating the association between hyponatremia and mortality in patients with HF.

**Table 3:** Cox regression analysis of hyponatremia and mortality outcomes in patients with heart failure. *: Significant at p-values <0.05. Cox regression models for mortality were adjusted for clinical parameters (LVEF, BMI, creatinine, eGFR). HR = hazard ratio; CI = confidence interval; LVEF = left ventricular ejection fraction; BMI = body mass index; eGFR = estimated glomerular filtration rate

Outcome variable	Sodium status (vs. normonatremia ≥135 mmol/L)	HR	95% CI	P-value
In-hospital mortality	Mild (130–134 mmol/L)	1.6	0.9–2.9	0.094
	Moderate (125–129 mmol/L)	2.2	1.1–4.1	0.021*
	Severe (<125 mmol/L)	3.1	1.5–6.4	0.002*
30-day mortality	Mild (130–134 mmol/L)	1.8	1.0–3.1	0.048*
	Moderate (125–129 mmol/L)	2.4	1.3–4.3	0.005*
	Severe (<125 mmol/L)	3.6	1.8–7.1	<0.001*

**Figure 1:**
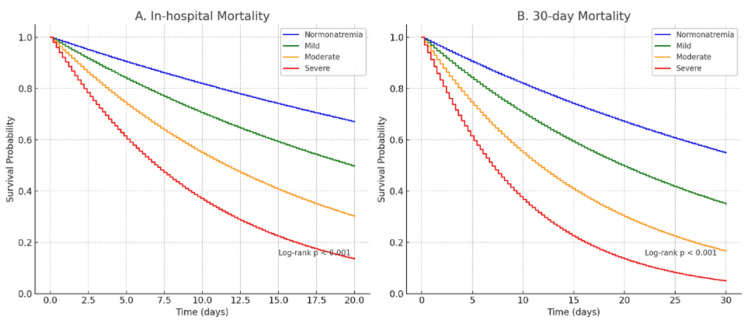
Kaplan-Meier survival curves.

The adjusted risk of in-hospital mortality and moderate (HR = 2.2, 95% CI = 1.145; p = 0.021) and severe hyponatremia (HR = 3.1, 95% CI = 1.546) were comparatively larger in normonatremic patients. The same gradient was observed with regard to 30-day mortality, with onset of HR = 1.8 in mild and HR = 3.6 in severe cases (p < 0.001). Kaplan-Meier survival curves for in-hospital and 30-day mortality stratified by hyponatremia severity on admission are shown in Figure 1.

Kaplan-Meier survival curves confirmed progressively lower survival with increasing hyponatremia severity for both in-hospital and 30-day outcomes. Log-rank tests showed significant differences across groups (p < 0.001). Proportional hazards assumptions were satisfied for all models (p > 0.05 by Schoenfeld residuals).

## Discussion

The present study aimed to evaluate the association between hyponatremia and clinical outcomes in hospitalized patients with HF. Our findings revealed that hyponatremia during admission was associated with in-hospital and 30-day mortality, length of stay, and impaired cardiac function. The underlying clinical decline linked to low serum sodium levels may be apparent in these laboratory abnormalities in HF patients. Thus, early detection of hyponatremia may aid medical professionals in identifying high-risk patients and directing prompt treatment plans [10].

Hyponatremic patients showed higher mortality rates and increased stays in hospitals than normonatremic patients. This finding is consistent with previous literature, which states that hyponatremia indicates high-grade neurohormonal stimulation, such as increased vasopressin and RAAS activity, hemodynamic impairment, later organ dysfunction, and fluid retention [11]. Other studies have found that serum sodium is linked to short- and long-term death in HF independently, which supports its use as an accessible prognostic biomarker [12]. Moreover, hyponatremia was negatively related to LVEF and positively related to increased BMI, indicating that patients with severe ventricular dysfunction and comorbid obesity are at a higher risk of sodium imbalance. Such associations can be attributed to the pathophysiological interaction of poor cardiac output, neurohormonal stimulation, and fluid imbalance in HF patients [13]. These mechanisms have supported the need to check the amount of sodium in the body as an overall HF treatment [14].

Even though some studies have demonstrated inconsistent outcomes with respect to interventions to correct hyponatremia [15], such as the careful management of fluids and vasopressin receptor antagonists [16], our results indicate that serum sodium should be assessed routinely on admission to the hospital. The variation in outcomes among studies could be related to variations in population characteristics, HF etiology, treatment regimens, and the time of sodium measurement [17]. However, hyponatremia is a simple, inexpensive, and biologically feasible disease severity and risk stratification in patients admitted to hospitals with HF [18]. Combined with other findings, these data indicate that hyponatremia is not a primary independent prognostic factor but an indication of the underlying pathophysiological load. This study used a prospective cohort study design with clear inclusion and exclusion criteria, which guaranteed diagnostic consistency, as suggested by ESC 2021 guidelines. Further, in-hospital and 30-day outcomes were compared, which provided short-term prognostic data on electrolyte imbalance in HF.

Although the sample size is limited, these aspects render the study more consistent and clinically relevant. The research was performed in one center and had a small sample size that could limit the extrapolation of findings. Moreover, the comorbidities were heterogeneous, and other possible confounders, including sodium intake in the diet, medication adherence, and neurohormonal changes in treatments, were not fully controlled. The observational study design prevents determining the cause and effect. Future multicentric, prospective, and randomized research is needed to determine whether the solution of hyponatremia improves the results or is just an indicator of the positive clinical stabilization. Sodium-guided longitudinal follow-up can help determine whether the early management of hyponatremia can reduce the mortality and readmission rates among HF patients.

## Conclusions

This study found that there was a strong correlation between admission hyponatremia and adverse short-term outcomes in patients hospitalized due to HF. Reduced serum sodium was linked with increased in-hospital and 30-day mortality, increased hospitalization, and poor cardiac function. These results support the usefulness of serum sodium as a simple and easily accessible prognostic factor in HF as a risk stratification tool. The timely diagnosis and treatment of hyponatremia can help physicians improve temporal outcomes, but the results need to be confirmed by larger multicenter studies.
